# A Potential Biomarker of Combination of Tumor Mutation Burden and Copy Number Alteration for Efficacy of Immunotherapy in *KRAS*-Mutant Advanced Lung Adenocarcinoma

**DOI:** 10.3389/fonc.2020.559896

**Published:** 2020-09-24

**Authors:** Luochengling Xiang, Xiao Fu, Xiao Wang, Wenyuan Li, Xiaoqiang Zheng, Kejun Nan, Tao Tian

**Affiliations:** ^1^Department of Oncology, First Affiliated Hospital of Xi’an Jiaotong University, Xi’an, China; ^2^Oncology Hospital, Xi’an International Medical Center Hospital, Xi’an, China

**Keywords:** *KRAS* mutation, lung adenocarcinoma, tumor mutation burden, copy number of alteration, biomarker

## Abstract

**Objectives:**

The *Kirsten Rat Sarcoma* (*KRAS*) mutation is the commonest oncogenic drive mutation in lung adenocarcinoma (LUAD) and immunotherapy may be quite promising for *KRAS*-mutant LUAD. While the effects of tumor mutation burden (TMB) and copy number alteration (CNA) are poorly understood in this illness, our study aimed to explore the roles TMB and CNA play in the prediction of response to immune checkpoint inhibitor (ICI) therapy in advanced *KRAS*-mutant LUAD.

**Methods:**

Mutation and clinical data were downloaded from cBioPortal. We evaluated *KRAS* mutation status and divided patients into different subgroups based on TMB and CNA cutoffs to investigate the predictive value of these biomarkers on ICI response.

**Results:**

*KRAS* mutation with concurrent *TP53* or *STK11* mutations had higher TMB and CNA compared to *KRAS* mutation alone. The *KRAS* G12C and G > T mutation subgroups, with *TP53* or *STK11* co-mutation, also had higher TMB and CNA. We found that TMB and CNA were independently associated with progression-free survival (PFS) and durable clinical benefits (DCB); TMB was positively correlated with PFS (*P* = 0.0074) and DCB (*P* = 0.0008) while low CNA was associated with prolonged PFS (*P* = 0.0060) and DCB (*P* = 0.0018). However, TMB alone did not distinguish benefits among *KRAS*-mutant patients. Notably, when combining TMB and CNA, low TMB and high CNA revealed worse outcomes of ICI therapy (mPFS: 2.20m, *P* = 0.0023; proportion of DCB: 24%, *P* = 0.0001).

**Conclusion:**

The combination of TMB and CNA provides more sensible and accurate prediction of ICI response than individual factors in *KRAS*-mutant LUAD. Moreover, low TMB and high CNA can be utilized as a potential biomarker to predict adverse outcome in *KRAS*-mutant LUAD.

## Introduction

In lung adenocarcinoma (LUAD), the most frequent oncogene driver mutation is *Kirsten Rat Sarcoma (KRAS)* (1). While patients harboring other driver genes, such as those for *Epidermal Growth Factor Receptor* (*EGFR)* and *Anaplastic Lymphoma Kinase (ALK)*, may respond to therapy with tyrosine kinase inhibitors (TKIs), those harboring a *KRAS* mutation lack efficient treatment regimens. Despite decades of research, the KRAS protein remains a challenging therapeutic target due to the lack of an ideal small molecule binding pocket in the protein and its high affinity toward the abundance of guanosine triphosphate (GTP). While several novel inhibitors targeting the mutant protein *KRAS* G12C (missense substitution at codon 12; glycine to cysteine) with covalent bonding to the cysteine amino acid have been used in early phase clinical trials, there are many *KRAS* mutation subtypes, such as G12V (missense substitution at codon 12; glycine to valine) and G12D (missense substitution at codon 12; glycine to aspartic acid) (2). Besides, although the *KRAS-MAPK* pathway is downstream of *EGFR* signaling, patients with a *KRAS* mutation do not respond to *EGFR* TKIs (3). In addition, patients with *KRAS*-mutant advanced non-small cell lung cancer (NSCLC) exhibit inferior responses to cytotoxic chemotherapy as well as decreased progression-free survival (PFS) and overall survival (OS) compared to patients harboring native *KRAS* (4). Recently, immunotherapy has become regarded as most promising for *KRAS*-mutant LUAD (5).

Immune checkpoint inhibitors (ICIs) have revolutionized the management of NSCLC. Treatment with anti-cytotoxic T lymphocyte antigen 4 (CTLA4) antibody and programmed cell death-1 (PD-1) or PD-1 ligand (PD-L1) inhibitors has greatly improved patient survival. Even though ICIs have emerged as epochal milestones in anti-cancer therapy, only a subset of patients exhibits objective responses and while others show disease progression. Patients treated with ICIs may also suffer life-threatening immune-related adverse effects and even suffer hyper progression of the disease (6). A detailed understanding of key predictive factors necessary to identify patients who may potentially benefit from treatment with ICIs is thus urgent.

To date, among patients with PD-L1-positive disease, tumor-infiltrating lymphocytes have proven to be indicators of ICI therapy (7, 8). Importantly, increasing evidence suggests that the diversity and composition of gut microbiota impacts patient response to ICIs (9, 10). Since the advent of next generation sequencing, an increasing number of genetic tumor features have also been detected, including tumor mutation burden (TMB), microsatellite instability and copy number alteration (CNA), which have been correlated with therapeutic response. The number of non-synonymous single nucleotide variants, or TMB, in a tumor was found to strongly positively correlate with response to ICIs in NSCLC (11, 12). However, Merkel cell carcinoma was reported to respond better than TMB alone expects, while colorectal carcinoma was found to have worse outcomes than that predicted by TMB alone (13). Interestingly, a pan-cancer analysis based on The Cancer Genome Atlas revealed a negative relationship between CNA and immune infiltration. Meanwhile, in the setting of anti-CTLA4 therapy, CNA was reported to be a potential predictive factor of survival, independent of TMB (14).

Here, to evaluate the potential utility of TMB and CNA together in identifying distinct patient subgroups of *KRAS*-mutant LUAD, we compared the distribution of TMB and CNA among different *KRAS* mutations and then analyzed efficacy of ICI treatment in subgroups based on TMB and CNA.

## Materials and Methods

### Clinical Cohorts

Data were collected from published articles. Mutation data of 860 advanced LUAD patients were retrieved from cBioPortal^1^. From this website, we obtained DNA sequencing data to analyze TMB and CNA distributions among multiple *KRAS* mutations. Details of samples included were shown as a flowchart in Supplementary Figure 1.

Clinical and mutation data of 240 NSCLC patients were also retrieved from cBioPortal^2^. We collected 186 advanced LUAD. All patients were treated with anti-PD-1/PD-L1 monotherapy or in combination with anti-CTLA4 blockade between April 2011 and January 2017. Details of these samples were also shown as a flowchart in Supplementary Figure 1. All patients had undergone the MSK-IMPACT assay, a next generation sequencing tumor profile test. Response Evaluation Criteria in Solid Tumors (RECIST) version 1.1 was performed to assess efficacy. Efficacy was additionally identified as durable clinical benefit (DCB; complete response (CR) or partial response (PR); or stable disease (SD) that lasted >6 months) or no durable benefit [NDB; progressive disease (PD) or SD that lasted ≤6 months]. Patient PFS was assessed from the date of immunotherapy initiation to the date of disease progression or death for any reason (15).

### Tumor Mutation Burden Analysis

Somatic mutation data of advanced LUAD were retrieved from cBioPortal. In the MSK-IMPACT assay, tumor and matched normal data were used to identify somatic variants and optimize mutation calling filters; 100× coverage was needed to defect mutations with true variant frequencies ≥10% with 98% power. All exons and selected introns of custom gene panels of 341 (version 1), 410 (version 2), and 468 (version 3) genes were sequenced and targeted. Patients were classified according to the coding region captured in each panel, thus covering 0.98, 1.06, and 1.22 megabases (Mb) in the 341-, 410-, and 468-gene panels, respectively. The TMB cutoff value was obtained using X-tile, a tool for outcome-based biomarker cut-point optimization (16).

### Copy Number Alteration Analysis

Data concerning CNA in the MSKCC database were analyzed by MSK-IMPACT sequencing. Via comparison of sequence coverage of targeted regions in a tumor sample with a standard normal sample, CNA was identified. The Genome Analysis Toolkit (GATK) was used to obtain coverage of targeted regions, and a Loess normalization was applied to adjust guanosine-cytosine content. Log-ratio coverage values were subsequently segmented by circular binary segmentation. Germline cells were removed to ensure somatic final copy number variants. Log_2_ copy number gain >0.2 or loss <−0.2 (*P* < 0.05) was used to determine significant whole gene gain or loss events (17).

### Statistical Analysis

Statistical analysis was conducted by Graph Prism (version 8.0) and SPSS (version 22.0). The Mann–Whitney U test was performed to compare TMB and CNA values; TMB and CNA were presented using box plots that presented mean, interquartile ranges, and ranges. Hazard ratio was determined via univariate and multivariate Cox proportional hazard regression analyses. Kaplan–Meier curve analysis was applied to evaluate PFS and OS using log-rank analysis. Proportional DCB representation was detailed by a 100% stacked column graph. Pearson’s Chi-squared test was applied to evaluate the difference in DCB proportion among different subgroups. All reported *P*-values were two-tailed, and for all analyses, *P* ≤ 0.05 was considered statistically significant.

## Results

### Prognostic Value of *KRAS* Mutation Status in Advanced Lung Adenocarcinoma

**FIGURE 1:**
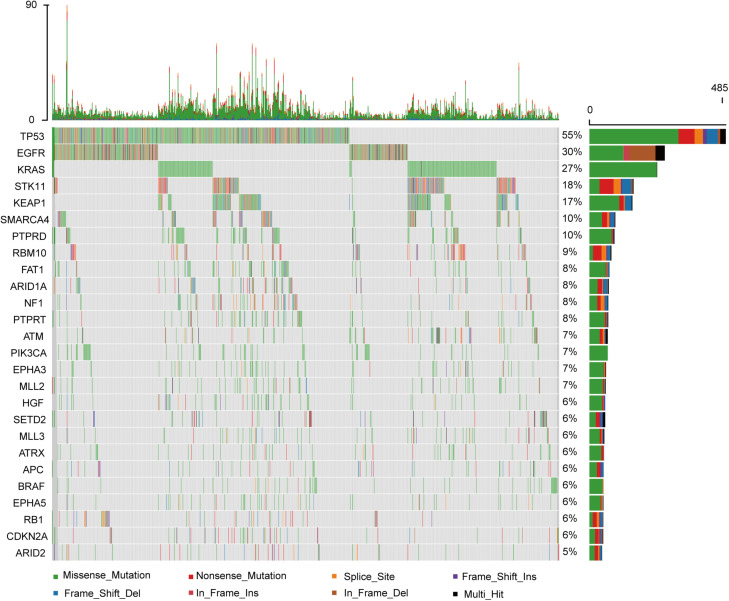
The genomic landscape and the mutation signature of advanced lung adenocarcinoma in the MSKCC database.

**FIGURE 2:**
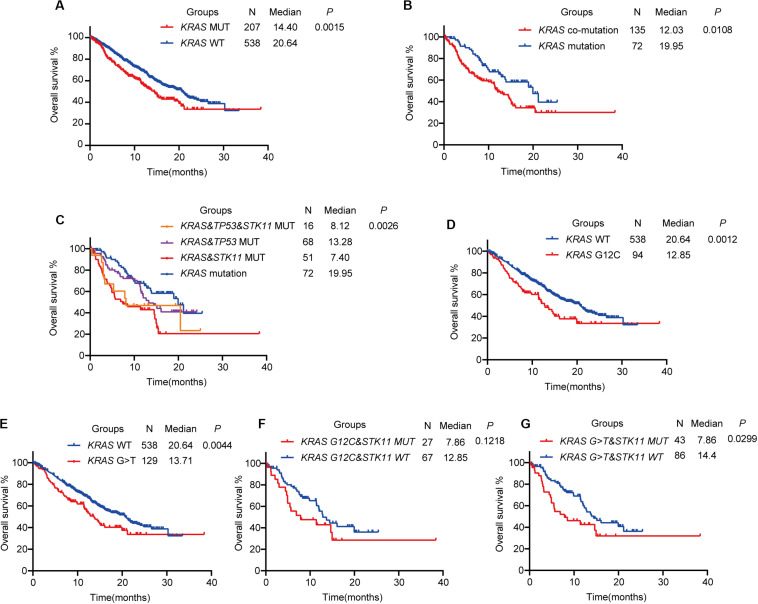
The prognostic value of *KRAS* mutational status in advanced lung adenocarcinoma. **(A)** Kaplan–Meier survival analysis based on *KRAS* mutation status. **(B)** *KRAS*-mutant patients with co-mutations have shorter overall survival than those with *KRAS* mutation alone. **(C)** Kaplan–Meier survival analysis of *KRAS* co-mutation subtypes. **(D,E)** Kaplan–Meier survival analysis of *KRAS* mutation subtypes G12C **(D)** or G > T **(E)** with wild-type. **(F,G)** Kaplan–Meier survival analysis of *KRAS* mutation subtypes G12C **(F)** or G > T **(G)** with concurrent *STK11* mutation. MUT, mutant; WT, wild-type.

Among the 860 metastatic LUAD patients who underwent genomic analysis in the MSKCC-IMPACT study (1), *KRAS* mutation was common (Figure 1). As shown in Supplementary Figure 1, we deleted 115 patients without matched survival data. A total of 207 patients with *KRAS* mutations had statistically shorter OS as compared with 538 patients with wild-type *KRAS* tumors (HR = 1.515; 95% CI: 1.172–1.960; *P* = 0.0015, Figure 2A).

The most common concurrent pathogenic mutations were *TP53* (84 patients, 40.6%) and *STK11* (67 patients, 32.4%), consistent with previous studies (18). We divided *KRAS*-mutant patients into two groups based on concurrent *TP53* and *STK11* mutation status. One group was the *KRAS* co-mutation group (*KRAS*-mutant patients with either *TP53* or *STK11* mutation) and the other was the *KRAS* mutation group (*KRAS-*mutant patients without *TP53* or *STK11* mutation). We found that patients in the *KRAS* co-mutation group had shorter OS than those in the *KRAS* mutation group (HR = 1.618; 95% CI: 1.128–2.505; *P* = 0.0108, Figure 2B). Further analysis revealed that *KRAS-*mutant patients with co-occurring *STK11* mutation had shorter OS than those with either co-occurring *TP53* (HR = 1.864; 95% CI: 1.115–3.117; *P* = 0.0176) or both *TP53* and *STK11* (HR = 2.856; 95% CI: 1.645–4.958; *P* = 0.0002) mutations. No significant difference between *KRAS-*mutant patients with and without co-occurring *TP53* and *STK11* mutations was noted (HR = 2.219; 95% CI: 0.886–5.555; *P* = 0.0234), likely because *KRAS-*mutant patients with co-occurring *TP53* and *STK11* mutations only totaled 16 (Figure 2C).

The *KRAS* G12C mutation (missense substitution at codon 12; glycine to cysteine) has been previously reported to be oncogenic and potentially targetable; several novel *KRAS* G12C inhibitors, such as AMG150 and MRTX849, are being studied (2). In advanced LUAD, the *KRAS* G12C mutation was the most common, accounting for 45.4% of all *KRAS*-mutant advanced LUAD (G12C: *N* = 94, 45.4%; G12V, missense substitution at codon 12; glycine to valine: *N* = 31, 15.0%; G12D, missense substitution at codon 12; glycine to aspartic acid: *N* = 28, 13.5%). At the same time, G > T substitution (nucleotide substitution in sequences coding for amino acids in protein; G is substituted by T, *N* = 129, 62.3%) was the most common nucleotide substitution in *KRAS*-mutant advanced LUAD. On Kaplan–Meier analysis, the *KRAS* G12C mutation subtype was associated with shorter OS than wild-type *KRAS* (HR = 1.741; 95% CI: 1.209–2.509; *P* = 0.0012, Figure 2D), as was the *KRAS* G > T mutation subtype (HR = 1.583; 95% CI: 1.154–2.170; *P* = 0.0044, Figure 2E). In further analysis of the effect of concurrent *STK11* mutation, the *KRAS* G12C mutation subtype with or without concurrent *STK11* mutation was not found to have significantly different OS (HR = 1.668; 95% CI: 0.872–3.190; *P* = 0.1218, Figure 2F). The *KRAS* G > T mutation subtype with co-occurring *STK11* mutation, however, was found to have a much shorter OS when compared to the co-occurring *STK11* mutation alone (HR = 1.869; 95% CI: 1.063–3.286; *P* = 0.0299, Figure 2G).

### Correlation Between *KRAS* Mutation and Tumor Mutation Burden in Advanced Lung Adenocarcinoma

**FIGURE 3:**
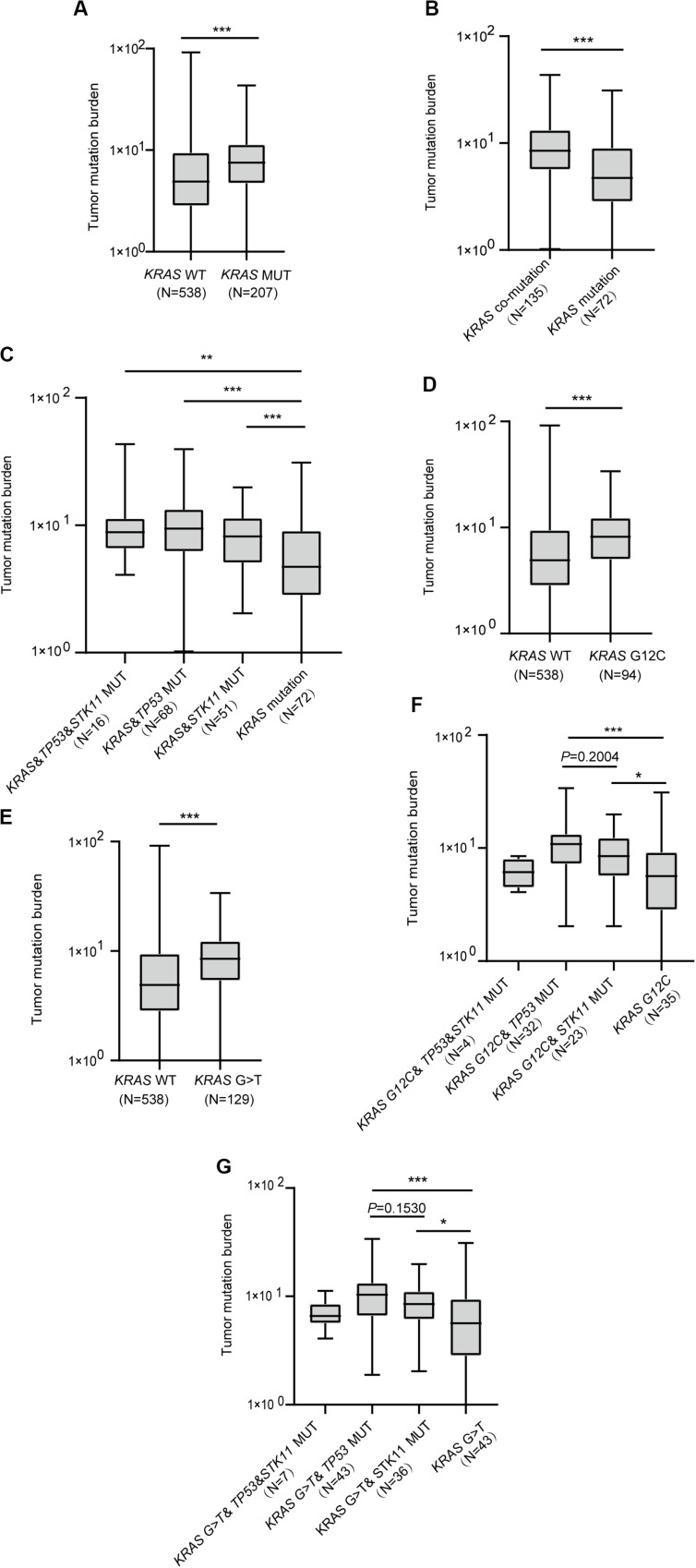
The correlation between *KRAS* mutational status and tumor mutation burden. **(A)** Patients with *KRAS* mutation have greater tumor mutation burden. **(B)** Patients with *KRAS* mutation and concurrent mutations have greater tumor mutation burden than those with *KRAS* mutation alone. **(C)** Comparison of tumor mutation burden in *KRAS* co-mutation subtypes. **(D,E)** Comparison of tumor mutation burden in *KRAS* G12C **(D)** and G > T **(E)** subtypes. **(F,G)** Comparison of tumor mutation burden in G12C **(F)** and G > T **(G)** subtypes with co-mutations. MUT, mutant; WT, wild-type. Box plot data are presented as mean, interquartile ranges, and ranges. ***P < 0.001; **P < 0.01; *P < 0.05.

Investigation of whether *KRAS* mutation status impacted TMB revealed significant differences in TMB among *KRAS* mutation and wild-type patients (*P* < 0.0001, Figure 3A). Moreover, patients with either *TP53* or *STK11* co-mutation had higher TMB than those with *KRAS* mutation alone (*P* < 0.0001, Figure 3B). Interestingly, each concurrent mutation was found to have higher TMB than *KRAS* mutation alone (*KRAS*&*TP53*&*STK11* vs. *KRAS*, *P* = 0.0023; *KRAS*&*TP53* vs. *KRAS*, *P* < 0.0001; *KRAS*&*STK11* vs. *KRAS*, *P* = 0.0005; Figure 3C).

Next, we sought to confirm the association between *KRAS* mutation subtypes and TMB. Results revealed that both *KRAS* G12C and G > T substitution mutations had higher TMB than did wild-type *KRAS* (*P* < 0.0001, Figure 3D; *P* < 0.0001, Figure 3E). We further found that *KRAS* G12C with either *TP53* or *STK11* co-mutation had higher TMB (*KRAS* G12C&*TP53* vs. *KRAS* G12C, *P* = 0.0005; *KRAS* G12C&*STK11* vs. *KRAS* G12C, *P* = 0.0264; Figure 3F). Similarly, *KRAS* G > T substitution mutation with either *TP53* or *STK11* co-mutation had higher TMB (*KRAS* G > T&*TP53* vs. *KRAS* G > T, *P* = 0.0004; *KRAS* G > T&*STK11* vs. *KRAS* G > T, *P* = 0.0129; Figure 3G).

### *KRAS* Mutation Status and Copy Number Alteration in Advanced Lung Adenocarcinoma

**FIGURE 4:**
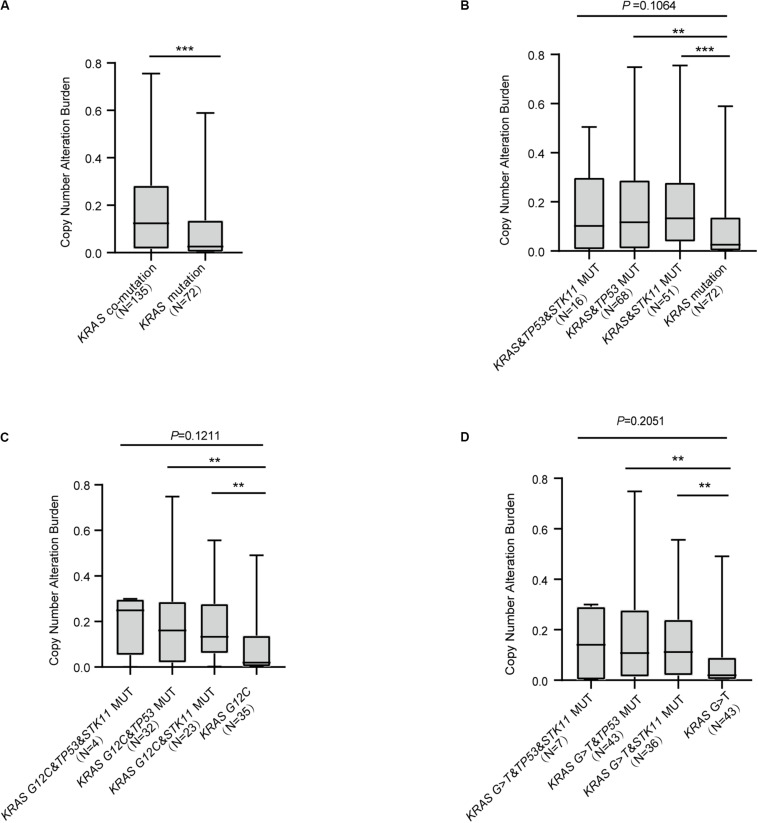
The correlation between *KRAS* mutational status and copy number alteration burden. **(A)** Patients with *KRAS* concurrent mutations have greater copy number alteration burden with only *KRAS* mutation. **(B)** Comparison of copy number alteration burden in *KRAS* co-mutation subtypes. **(C,D)** Comparison of copy number alteration burden in *KRAS* G12C **(C)** or G > T **(D)** subtypes with concurrent mutations. MUT, mutant; WT, wild-type. Box plot data are presented as mean, interquartile ranges, and ranges. ***P < 0.001; **P < 0.01.

Recent studies have reported CNA to be useful in the construction of predictive models concerning response to ICI treatment (13, 14). Our analysis revealed that *KRAS* mutation with concurrent mutations had higher CNA compared with *KRAS* mutation alone (*P* < 0.0001, Figure 4A). We further found that *KRAS* mutation with either *TP53* or *STK11* co-mutation significantly differed in CNA (*KRAS*&*TP53* vs. *KRAS* mutation, *P* = 0.0021; *KRAS*&*STK11* vs. *KRAS* mutation, *P* = 0.0002; Figure 4B). Analysis of the relationship between the common *KRAS* G12C and G > T substitution mutation subtypes and CNA revealed similar findings; both subtypes with either *TP53* or *STK11* co-mutation had significant differences in CNA (*KRAS* G12C&*TP53* vs. *KRAS* G12C, *P* = 0.0014; *KRAS* G12C&*STK11* vs. *KRAS* G12C, *P* = 0.0029; Figure 4C; *KRAS* G > T&*TP53* vs. *KRAS* G > T, *P* = 0.0022; *KRAS* G > T&*STK11* vs. *KRAS* G > T, *P* = 0.0015; Figure 4D).

### Independent Predictive Value of Tumor Mutational Burden and Copy Number Alteration for Immune Checkpoint Inhibitor Response in Advanced Lung Adenocarcinoma

**FIGURE 5:**
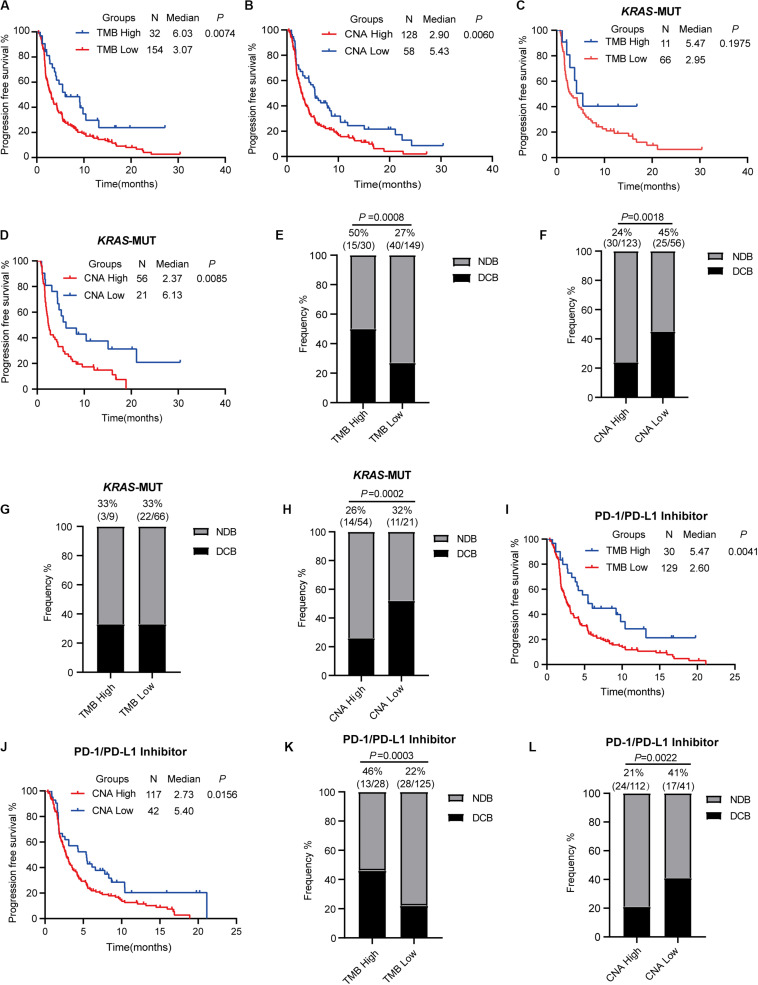
Tumor mutation burden and copy number alteration burden correlated with clinical response to immune checkpoint inhibitor treatment. **(A,B)** Progression-free survival curve for patients receiving ICI (PD-1/L1 inhibitor or in combination with anti-CTLA-4) based on tumor mutation burden **(A)** or copy number alteration burden **(B)**. **(C,D)** Progression-free survival curve for *KRAS*-mutant patients receiving ICI (PD-1/L1 inhibitor or in combination with anti-CTLA-4) based on tumor mutation burden **(C)** and copy number alteration burden **(D)**. **(E,F)** Proportional representation of durable clinical benefits in advanced lung adenocarcinoma patients receiving ICI (PD-1/L1 inhibitor or in combination with anti-CTLA-4). **(G,H)** Proportional representation of durable clinical benefits in advanced *KRAS*-mutant lung adenocarcinoma patients receiving ICI (PD-1/L1 inhibitor or in combination with anti-CTLA-4). **(I,J)** Progression-free survival curve for patients receiving PD-1/PD-L1 inhibitor alone based on tumor mutation burden **(I)** and copy number alteration burden **(J)**. **(K,L)** Proportional representation of durable clinical benefits in advanced *KRAS*-mutant lung adenocarcinoma patients receiving PD-1/PD-L1 inhibitor alone. MUT, mutant; WT, wild-type; DCB, durable clinical benefit; NDB, no durable clinical benefit.

To estimate the predictive value of TMB and CNA in patient response to ICI treatment, available data in the MSKCC database were analyzed. A total of 240 patients with advanced NSCLC who underwent PD-1/PD-L1 inhibitor treatment alone or in combination with anti-CTLA-4 treatment were identified (15). We chose 186 patients with advanced LUAD for further analysis. For this particular population with ICI (PD-1/PD-L1 inhibitor alone or in combination with anti-CTLA-4), optimal cutoff points for TMB (13.27 mut/Mb) and CNA (0.05) were acquired using X-tile software. This population was subsequently divided into high (TMB ≥ 13.27 mut/Mb) and low (TMB < 13.27 mut/Mb) TMB groups; high TMB group patients were found to have significantly prolonged PFS (HR = 0.596; 95% CI: 0.408–0.870; *P* = 0.0074, Figure 5A) as well as an increased proportion of DCB (50 vs. 27%, *P* = 0.0008, Figure 5E). Analysis of patients classified into high (CNA ≥ 0.05) and low (CNA < 0.05) CNA groups revealed high CNA to be associated with shortened PFS (HR = 1.578; 95% CI: 1.140–2.184; *P* = 0.0060, Figure 5B) and a decreased proportion of DCB (24 vs. 45%, *P* = 0.0018, Figure 5F). Cox proportional hazard regression analysis revealed, after multivariate adjustment, TMB and CNA to be independent biomarkers for ICI response (TMB, HR = 0.46, *P* = 0.0011; CNA, HR = 1.86, *P* = 0.0007, Table 1).

**TABLE 1:** Univariable and multivariable Cox proportional hazards regression.

	Univariable analysis				Multivariable analysis			
		95%CI				95%CI		
Variable	HR	Lower	Upper	*P*	HR	Lower	Upper	*P*
Age	1.00	0.99	1.01	0.942				
Gender (male vs. female)	1.04	0.76	1.42	0.809				
Smoker (yes vs. no)	0.74	0.51	1.06	0.103				
TMB (≥13.27 mut/Mb vs. <13.27 mut/Mb)	0.54	0.34	0.85	0.008	0.46	0.29	0.73	0.0011
CNA (≥0.05 vs. <0.05)	1.63	1.15	2.31	0.007	1.86	1.30	2.66	0.0007

*HR, hazard ratio; CI, confidence interval.*

We evaluated the data of 77 *KRAS-*mutant patients from the population outlined above to further confirm our findings, but no significant differences in PFS (HR = 0.636; 95% CI: 0.319–1.266; *P* = 0.1975, Figure 5C) and proportion of DCB (high vs. low TMB; 33 vs. 33%, Figure 5G) were noted in the *KRAS*-mutant population. Significantly prolonged PFS (HR = 0.497; 95% CI: 0.293–0.837; *P* = 0.0085, Figure 5D) and higher proportion of DCB (high vs. low CNA; 26 vs. 52%, *P* = 0.0002, Figure 5H) were observed in *KRAS*-mutant patients of the low CNA group as compared to those in the high CNA group.

Recent studies revealed high TMB to be correlated with combination PD-1 and CTLA-4 inhibitor treatment efficacy in NSCLC (11, 19). However, the predictive value of TMB in PD-1/PD-L1 inhibitor efficacy in patients with advanced NSCLC remains uncertain. We classified 159 advanced LUAD patients treated with anti-PD-1/PD-L1 monotherapy into two (high and low TMB) groups using a TMB cutoff value of 13.27 mut/Mb. Our findings revealed that high TMB was significantly correlated with prolonged PFS and greater DCB (HR = 0.564; 95% CI: 0.382–0.834; *P* = 0.0041, Figure 5I; DCB, 46 vs. 22%, *P* = 0.0003, Figure 5K). We found that low CNA was also associated with prolonged PFS and greater DCB (median PFS in high vs. low CNA group patients, 2.73 vs. 5.40 months, *P* = 0.0156, Figure 5J; DCB, 21 vs. 41%, *P* = 0.0022, Figure 5L).

### Low Tumor Mutational Burden and High Copy Number Alteration Together Predict a Poor Response to Immune Checkpoint Inhibitor Therapy

**FIGURE 6:**
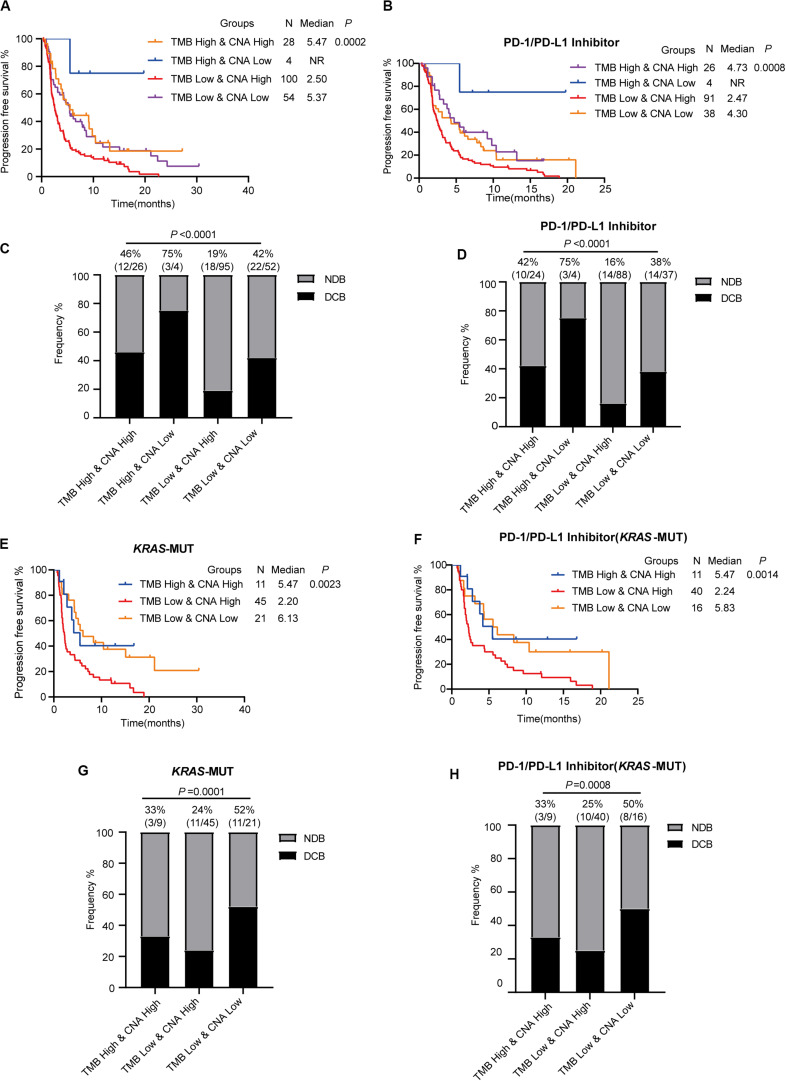
Low tumor mutational burden and high copy number alteration together predict a poor response to immune checkpoint inhibitor therapy. **(A,B)** Low TMB and high CNA show shorter progression-free survival in patients receiving ICI (PD-1/L1 inhibitor or in combination with anti-CTLA-4) **(A)** and patients receiving PD-1/PD-L1 inhibitor alone **(B)**. **(C,D)** Low TMB and high CAN show decreased proportion of DCB in patients receiving ICI (PD-1/L1 inhibitor or in combination with anti-CTLA-4) **(C)** and patients receiving PD-1/PD-L1 inhibitor alone **(D)**. **(E,F)** Low TMB and high CNA show shorter progression-free survival in *KRAS-*mutant patients receiving ICI (PD-1/L1 inhibitor or in combination with anti-CTLA-4) **(E)** and *KRAS-*mutant patients receiving PD-1/L1 inhibitor alone **(F)**. **(G,H)** Low TMB and high CNA show decreased proportion of DCB in *KRAS-*mutant patients receiving ICI (PD-1/L1 inhibitor or in combination with anti-CTLA-4) **(G)** and *KRAS-*mutant patients receiving PD-1/L1 inhibitor alone **(H)**. MUT, mutant; WT, wild-type; DCB, durable clinical benefit; NDB, no durable clinical benefit.

As TMB and CNA were established independent predictive factors of ICI response, we conjectured that combined use of both TMB and CNA would better predict ICI efficacy. In advanced LUAD patients with ICI (PD-1/PD-L1 inhibitor alone or in combination with anti-CTLA-4), low TMB and high CNA were found to have significantly shorter PFS compared to patients with high TMB and high CNA, high TMB and low CNA, and low TMB and low CNA (low TMB and high CNA vs. high TMB and high CNA: HR = 1.803, 95% CI: 1.199–2.712, *P* = 0.0047; low TMB and high CNA vs. high TMB and low CNA: HR = 2.693, 95% CI: 1.276–5.683, *P* = 0.0094; low TMB and high CNA vs. low TMB and low CNA: HR = 1.752, 95% CI: 1.240–2.476, *P* = 0.0015; Figure 6A). Patients with low TMB and high CNA had the significantly lowest proportion of DCB as compared to those in the three aforementioned subgroups (low TMB and high CNA vs. high TMB and high CNA vs. high TMB and low CNA vs. low TMB and low CNA; 19 vs. 46 vs. 75 vs. 42%, *P* < 0.0001, Figure 6C). Our analysis revealed findings consistent with those above in advanced LUAD patients with PD-1/PD-L1 inhibitor alone; patients with low TMB and high CNA were confirmed to have the significantly shortest PFS (low TMB and high CNA vs. high TMB and high CNA: HR = 1.771, 95% CI: 1.156–2.713, *P* = 0.0086; low TMB and high CNA vs. high TMB and low CNA: HR = 2.851, 95% CI: 1.385–5.872, *P* = 0.0045; low TMB and high CNA vs. low TMB and low CNA: HR = 1.608, 95% CI: 1.095–2.363, *P* = 0.0154, Figure 6B) and lowest proportion of DCB (low TMB and high CNA vs. high TMB and high CNA vs. high TMB and low CNA vs. low TMB and low CNA: 16 vs. 42 vs. 75 vs. 38%, *P* < 0.0001, Figure 6D).

Next, we further analyzed the predictive value of low TMB and high CNA in *KRAS*-mutant LUAD. In those patients with ICI (PD-1/PD-L1 inhibitor alone or in combination with anti-CTLA-4), although there were no *KRAS*-mutant LUAD patients in the high TMB and low CNA subgroup, patients with low TMB and high CNA were found to have shortened PFS (low TMB and high CNA vs. high TMB and high CNA: HR = 1.977, 95% CI: 1.025–3.814, *P* = 0.0420; low TMB and high CNA vs. low TMB and low CNA: HR = 2.338, 95% CI: 1.368–3.995, *P* = 0.0019, Figure 6E) and a smaller proportion of DCB (low TMB and high CNA vs. high TMB and high CNA vs. low TMB and low CNA: 24 vs. 33 vs. 52%, *P* = 0.0001, Figure 6G). Significant differences in PFS (low TMB and high CNA vs. high TMB and high CNA: HR = 1.994, 95% CI: 1.021–3.894, *P* = 0.0433; low TMB and high CNA vs. low TMB and low CNA: HR = 2.022, 95% CI: 1.131–3.616, *P* = 0.0176, Figure 6F) and DCB (low TMB and high CNA vs. high TMB and high CNA vs. low TMB and low CNA: 25 vs. 33 vs. 50%, *P* = 0.0008, Figure 6H) in patients with low TMB and high CNA receiving anti-PD-1/PD-L1 monotherapy were noted compared with those of the other two groups. Thus, the combination of TMB and CNA was confirmed to increase the sensitivity of ICI efficacy prediction in advanced *KRAS*-mutant LUAD. In addition, the combination of low TMB and high CNA was confirmed to predict poor ICI response in advanced *KRAS*-mutant LUAD.

## Discussion

Among lung cancer patients, *KRAS* mutation is the commonest mutation and 27% of LUAD patients harbor it (20). Patients suffering *KRAS*-mutant NSCLC continue to have a poor prognosis and lack efficient treatment strategies. Effective pharmacologic targeting of *KRAS* mutations also remains an unprecedented challenge. Recent studies, however, have reported that patients suffering *KRAS*-mutant NSCLC treated with ICI therapy had improved OS and PFS compared to those treated with chemotherapy (21, 22). In addition, TMB and CNA have been reported to be features of the genomic landscape that affect ICI efficacy (13). Here, we found that combined use of TMB and CNA increased the predictive sensitivity for ICI response in patients suffering *KRAS*-mutant advanced LUAD. Importantly, we found that low TMB and high CNA were associated with a poor prognosis, and TMB level positively correlated with response to anti-PD-1/PD-L1 monotherapy.

Recent studies have reported *KRAS*-mutant tumors to show greater PD-L1 expression (23) and T-cell infiltration (24). Here, our analysis of the correlation between KRAS mutation status and TMB revealed TMB to be associated with tumor immunogenicity and greater benefit of ICI therapy (25). We found that *KRAS*–mutant tumors showed higher TMB than did wild-type tumors. In further analysis of mutation subtypes and co-mutations, we demonstrated that *KRAS* with either co-occurring *TP53* or *STK11* mutation had greater TMB as compared to *KRAS* mutation alone. In *KRAS*-mutant LUAD, *KRAS* with *STK11* co-mutation was reported to facilitate immune escape and resistance to anti-PD-1 therapy and to mostly be an “immune desert” phenotype (26, 27). Interestingly, *TP53* inactivation in *KRAS*-mutant LUAD was reported to increase inflammatory marker levels and improve PFS (21, 27).

Tumor CNA burden has been reported to be a pan-cancer prognostic factor for recurrence and death (28). Here, we found that *KRAS* with either co-occurring *TP53* or *STK11* mutation had higher CNA. Furthermore, high CNA was a potential predictor of poor ICI efficacy in *KRAS*-mutant advanced LUAD. This finding was in agreement with prior evidence of CNA as a biomarker predictive for ICI response. Recently, CNA was reported to improve cell proliferation, reduce immune infiltration, and at lower levels correlate with poor ICI response (14). Of note, CNA likely is involved in the suppression of antigen presentation in cancer cells (29).

Although TMB and CNA have been reported to impact immune infiltration and predict ICI response, there have been few studies exploring associations among the combined application of TMB and CNA and clinical benefits of ICI. Multivariate Cox proportional hazard regression analysis of TMB and CNA confirmed that these two biomarkers were independent predictive factors for ICI response. Thus, while CNA provides complementary analysis of clinical ICI response, combining TMB and CNA improves the predictive sensitivity and accuracy of ICI response compared to use of these biomarkers independently. We divided patients into subgroups based on the cutoff value of TMB (13.27 mut/Mb) and CNA (0.05) from X-tile software. Previous studies have revealed that a cut-off value for TMB of 14.31 mut/Mb was used to predict survival in patients who underwent immunotherapy for advanced gastric cancer (30), while intermediate CNA was found to discriminate for recurrence in a prostate cancer population (31). Therefore, more researches are needed to speculate the optimal cutoff for clinical practices. We found that patients with low TMB and high CNA suffered significantly worse outcomes in the setting of ICI therapy. In *KRAS*-mutant LUAD, combination of TMB and CNA revealed that patients with low TMB and high CNA suffered a significantly worse prognosis. Thus, combined application of TMB and CNA values can be used to accurately select patients who would benefit from ICI treatment.

Our research had several limitations. First, all of our data were obtained from open databases, and patient characteristics were limited. As such, we were confined to analyzing data that was available. For example, patients receiving ICI treatment had PFS but lacked OS data; thus we could only analyze differences in PFS. In addition, we were only able to obtain genomic and clinical data; as PD-L1 mRNA expression and TPS data were unavailable, we could not compare any difference among them across *KRAS*-mutant LUAD subgroups. Finally, as our analysis was retrospective in nature, prospective and multi-center clinical trials should further be performed prior to utilization of combined TMB and CNA in the prediction of patient outcomes to ICI therapy.

## Conclusion

In conclusion, we here detailed that combining TMB and CNA provides a potential biomarker that effectively predicts patient response to ICI therapy. We found that TMB and CNA were higher in *KRAS*-mutant tumors as compared to wild-type tumors. Furthermore, *KRAS* with either *TP53* or *STK11* co-mutations had higher TMB and CNA as compared with *KRAS* alone. Our findings highlight that low TMB and high CNA is useful in predicting adverse patient outcomes for ICI therapy.

## Data Availability Statement

The datasets presented in this study can be found in online repositories. The names of the repository/repositories and accession number(s) can be found in the article/ Supplementary Material.

## Ethics Statement

The studies involving human participants were reviewed and approved by the Medical Ethics Committee of Xi’an Jiaotong University. The patients/participants provided their written informed consent to participate in this study.

## Author Contributions

LX, XF, KN, and TT designed the study and wrote the manuscript. XW, WL, and XZ downloaded and analyzed the data. All authors contributed to the article and approved the submitted version.

## Conflict of Interest

The authors declare that the research was conducted in the absence of any commercial or financial relationships that could be construed as a potential conflict of interest.

## Footnotes

1 https://www.cbioportal.org/study/summary?id=lung_msk_2017

2 https://www.cbioportal.org/study/summary?id=nsclc_pd1_msk_2018

## Supplementary Material

Supplementary Figure 1 Flowchart of study. TMB, tumor mutation burden; CNA, copy number alteration; MUT, mutant; WT, wild-type; DCB, durable clinical benefit; NDB, no durable clinical benefit.
