# Supplementary figures and images for: A Potential Biomarker of Combination of Tumor Mutation Burden and Copy Number Alteration for Efficacy of Immunotherapy in KRAS-Mutant Advanced Lung Adenocarcinoma
Source: Front Oncol. 2020 Sep 24;10:559896. doi: 10.3389/fonc.2020.559896 (PMC7541961; doi:10.3389/fonc.2020.559896)

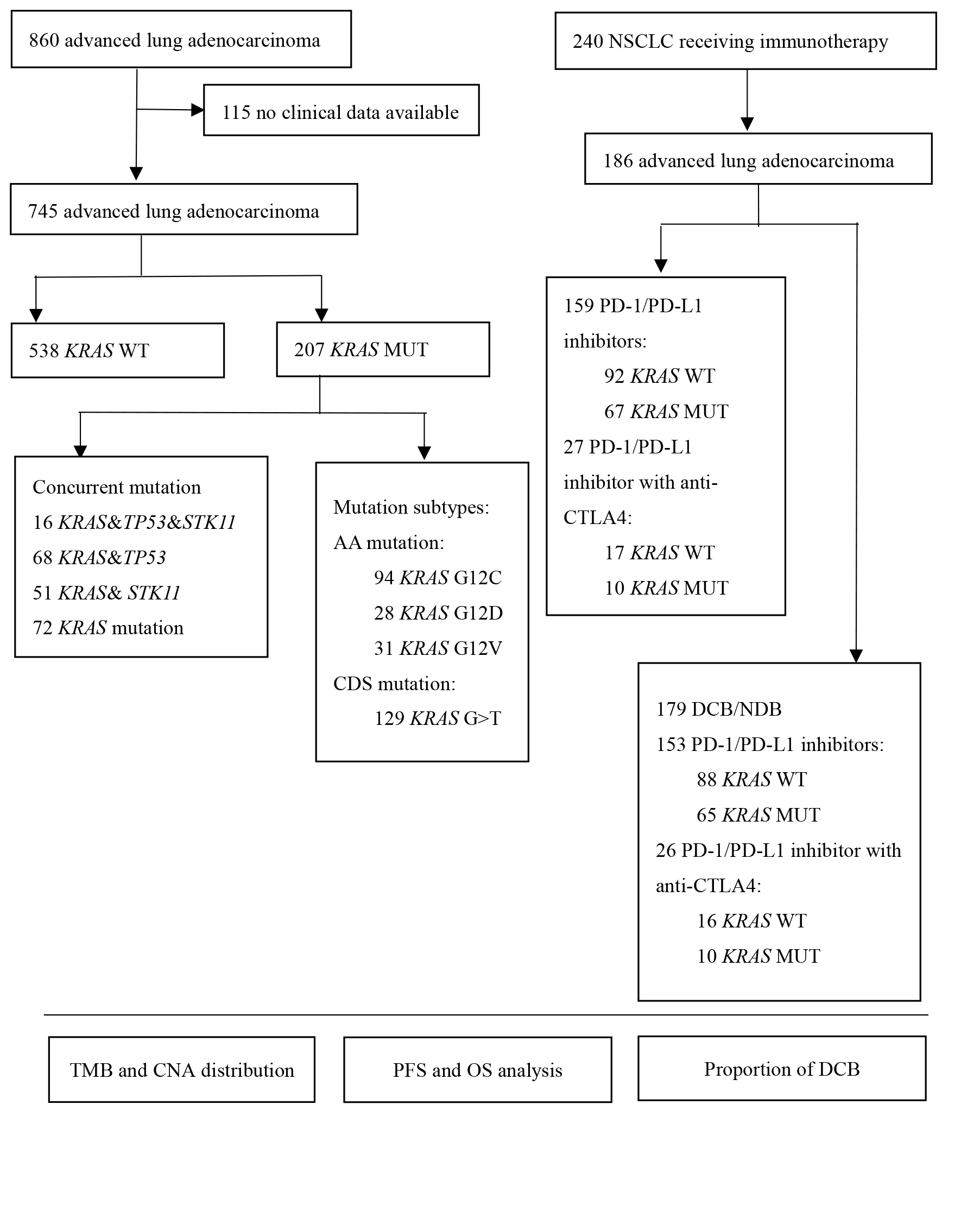

Supplement: Supplementary Figure 1 — Flowchart of study. TMB, tumor mutation burden; CNA, copy number alteration; MUT, mutant; WT, wild-type; DCB, durable clinical benefit; NDB, no durable clinical benefit. [file Image_1.TIF]
